# Spatial enrichment of PTPRZ1 in the peritumoral niche drives glioblastoma migration via the OPA1/ROS/CDH2 axis

**DOI:** 10.1186/s12967-026-08884-7

**Published:** 2026-08-26

**Authors:** Li Yi, Shuhang Qin, Min Guo, Xuya Wang, Yiming Zhang, Peiquan Guo, Arne Östman, Xuejun Yang, Xiaobing Jiang

**Affiliations:** 1 Department of Neurosurgery, Union Hospital, Tongji Medical College, Huazhong University of Science and Technology, Wuhan, 430022 China; 2 Department of Oncology-Pathology, Karolinska Institutet, BioClinicum, Solna, Stockholm 17164 Sweden; 3 Department of Obstetrics and Gynecology, The First Affiliated Hospital, Sun Yat-sen University, Guangzhou, 510080 China; 4 Department of Radiology, Beijing Tiantan Hospital, Capital Medical University, Beijing, 100070 China; 5 Department of Neurosurgery, Beijing Tsinghua Changgung Hospital, Beijing, 102218 China; 6 Department of Neurosurgery, Tianjin Medical University General Hospital, Tianjin, 300052 China

## Abstract

**Background:**

Glioblastoma (GBM) is characterized by high invasiveness and metabolic heterogeneity. Protein Tyrosine Phosphatase Receptor Type Z1 (PTPRZ1) has been implicated in glioma stemness and tumor grade progression, while the molecular mechanism remains unknown.

**Methods:**

Integrated bioinformatics analysis of TCGA dataset and TMA IHC profiling were used to explore clinical relevance of PTPRZ1, and potential molecular mechanisms of PTPRZ1 involved glioma progression. Ivy Glioblastoma datasets analysis and multiregional GBM IHC were performed to spatially characterize the expression patterns of PTPRZ1 in GBM tissues. Zebrafish xenograft model was established to elucidate the role of PTPRZ1 in driving glioblastoma cell migration. Western blotting, mitochondrial functional assays, Transwell migration assays, and human GBM tissue multiplexing were employed to investigate the mechanism of PTPRZ1 mediated glioma cell mobility via maintaining mitochondrial functions.

**Results:**

Spatial profiling revealed that PTPRZ1 is significantly enriched in the infiltrating areas and peritumoral margins of GBM, and acts as a poor prognosis factor in lower grade astrocytoma, proneural and mesenchymal GBM. Mechanistically, PTPRZ1 expression was found to be strongly correlated with cell adhesion molecules (CAMs) and mitochondrial metabolism across multiple glioma subtypes. Knockdown of PTPRZ1 triggered a shift from elongated to fragmented mitochondrial morphology by specifically downregulating the mitochondrial fusion protein OPA1. This mitochondrial dysfunction led to a metabolic shift toward glycolysis and a marked accumulation of ROS, which suppressed N-cadherin expression, thereby impairing glioma cell migration. These findings were corroborated in vivo, where PTPRZ1 depletion significantly reduced disseminated tumor foci in a zebrafish model.

**Conclusion:**

Our study demonstrates that PTPRZ1 drives glioblastoma migration by sustaining an OPA1-dependent mitochondrial fusion program, which suppresses mitochondrial ROS levels and subsequently stabilizes a pro-migratory phenotype via N-cadherin expression. Targeting the PTPRZ1/OPA1/ROS axis represents a promising therapeutic strategy to inhibit the invasive expansion of GBM cells.

**Supplementary Information:**

The online version contains supplementary material available at https://doi.org/10.1186/s12967-026-08884-7.

## Background

Gliomas represent the most common form of primary brain tumors in adults and the most malignant form, glioblastoma, presents with heterogeneous morphology and resistance to chemotherapy [1]. To date, treatment decisions rely mainly on histologic classification which divides diffuse gliomas into oligodendroglioma, oligoastrocytoma, astrocytoma and glioblastoma (GBM), ranging from WHO grade 2 to 4 [2]. However, this partition method is highly subjective due to interobserver variability and inconsistent designation of glioma subtypes and grades [3, 4]. Recent molecular characterization studies have benefited from the availability of the datasets generated by The Cancer Genome Atlas (TCGA) [5–7] and genomic features, such as isocitrate dehydrogenase gene (IDH1/2) mutations, 1p/19q codeletion and histone H3‑K27M mutation were incorporated into the diagnostic criteria in the 2016 WHO classification [8, 9].

Diffuse gliomas can also be subdivided into several classes based on molecular criteria. Lower grade (Grade 2–3) gliomas and secondary glioblastomas (arising from lower grade gliomas) display IDH1/2 mutations in 80% of the cases [10]. Primary glioblastomas (originating de novo) lack IDH1 and IDH2 mutations. The presence of these mutations is now often used as an independent predictor of prolonged survival. Based on transcriptional profiles, glioblastomas are further subdivided into proneural (PN), mesenchymal (MES) and classical (CL) subtypes [11]. A subset of proneural glioblastomas display IDH1/2 mutations, suggesting a close relationship with lower grade glioma (LGG) and a better prognosis compared to that of other subtypes of GBM [9]. Constitutively active mutant versions of receptor tyrosine kinases (RTKs), e.g. EGFR (EGFRvIII), PDGFRA or MET, are commonly discovered in primary glioblastomas [12–14], underscoring deregulated phosphotyrosine-based signaling as a major driver in glioblastoma development [15]. However, compared to RTKs, the role of the counter-acting receptor tyrosine phosphatase (RTP) family in gliomas is much less studied.

Protein Tyrosine Phosphatase, Receptor Type Z1(PTPRZ1), as a member of the RTP family, is predominantly expressed in the central nervous system (CNS). PTPRZ1 located in the N-terminal region in the membrane, interacts with its ligand pleiotrophin (PTN), which is a secreted growth factor involved in angiogenesis and tumor growth as well as in CNS development [16, 17]. Upon binding, PTN inactivates the phosphatase activity of PTPRZ1, thereby augmenting phosphoprotein-dependent signalings and molecules, such as the Wnt/β-catenin pathway, Fyn and p190 RhoGAP [18–21]. Studies have demonstrated that PTN and PTPRZ are both highly expressed in oligodendrocyte precursor cells (OPCs) in human brain white matter. PTPRZ1 signaling contributes to the homeostatic self-renewal of OPCs mediated by the tonic activation of β-catenin/TCF-dependent transcription, and PTN suppression of PTPRZ1 promotes remyelination through the activation of OPC differentiation [22, 23].

PTPRZ1 is strongly expressed in malignant gliomas especially in glioma stem cells. It was reported that tumor-associated macrophages could secrete PTN to promote PTPRZ1 signaling in glioblastoma stem cells for tumor growth [24]. In addition, neural precursor cell-derived PTN mediates subventricular zone invasion of glioma [25, 26]. Importantly, studies have demonstrated a novel, frequent PTPRZ1-MET fusion in secondary glioblastoma, which could enhance MET signaling activity and contributes to a malignant phenotype and temozolomide resistance of glioblastoma [27–30]. However, comprehensive studies on PTPRZ1 in gliomas are still lacking.

In the present study, we demonstrated that PTPRZ1 is significantly upregulated in glioblastoma (GBM) and serves as a robust prognostic biomarker associated with diminished overall survival in glioma patients. This clinical relevance is particularly pronounced within the astrocytoma, Proneural, and Mesenchymal subtypes of GBM.

Functional enrichment analysis of PTPRZ1-positive related gene signatures revealed a potent correlation between PTPRZ1 and key cell adhesion molecules, most notably N-cadherin (CDH2). Spatially, PTPRZ1 was found to be preferentially enriched in the infiltrative margins of GBM tumors. Accordingly, genetic knockdown of PTPRZ1 markedly suppressed GBM cell metastasis in a zebrafish xenograft model.

From a metabolic perspective, PTPRZ1 deficiency led to a significant reduction in OPA1 expression, shifting mitochondrial dynamics toward extensive fission and triggering an elevation in reactive oxygen species (ROS) production. This metabolic shift effectively abrogated cell motility by inhibiting N-cadherin expression. Furthermore, a strong co-expression pattern among PTPRZ1, OPA1, and N-cadherin was validated within the intratumoral regions of clinical GBM tissues.

In conclusion, our findings identify PTPRZ1 as a critical pro-tumorigenic driver that promotes GBM infiltration by maintaining OPA1-mediated mitochondrial fusion. By stabilizing mitochondrial function and limiting ROS accumulation, PTPRZ1 sustains OPA1/N-cadherin-dependent migratory programs, highlighting its potential as a therapeutic target to combat GBM invasion.

## Methods and materials

### Gene expression profile and datasets

Pan cancer mRNA expression profile was accessed from GEPIA (http://gepia.cancer-pku.cn/). The mRNA expression profile and clinical prognosis data were obtained from The Cancer Genome Atlas (TCGA) database (https://cancergenome.nih.gov/), which includes 530 cases of Illumina RNA Sequence LGG samples and 539 cases of AffyU133a microarray GBM samples data. The mRNA expression profile of five structures (Leading Edge, Infiltrating Tumor, Cellular Tumor, Microvascular Proliferation, and Pseudopalisading Cells Around Necrosis) within GBM tissues were available from Ivy Glioblastoma Atlas Project (http://glioblastoma.alleninstitute.org/).

### Clinical sample collection

Glioma tissue microarray and tissue whole sections were obtained from the Department of Neurosurgery at Tianjin Medical University General Hospital. All the samples were histologically graded in accordance with the 2016 World Health Organization (WHO) classification for brain tumors by pathologists. The Ethical Committee of TMUGH granted approval for experiments on human glioma tissues.

### Tissue microarrays and immunohistochemical staining

The cases included in the tissue microarrays (TMA) and the immunohistochemical staining were previously indicated [31]. Sections were incubated sequentially with the anti-PTPRZ1 antibodies (#34977, SAB, USA) at 4˚C overnight. The next day, after rewarming for 1 h, sections were incubated with secondary antibodies (ZSGB-Bio, China) for 1 h at 37 °C. Immunostaining was performed using two-steps detection kit (ZSGB-Bio, China), which resulted in a brown precipitate at the antigen site. Subsequently, sections were counterstained with Mayer Haematoxylin solution (ZSGB-Bio, China) and mounted in mounting medium. The primary antibody was omitted for the negative controls. After dehydration, sections were examined using a microscope scanner. Protein was semi-quantified (8 levels) on the basis of the average staining extent and intensity. PTPRZ1 expression was quantified using a traditional semi-quantitative immunoreactivity score (IRS = intensity × percentage; range 0–12). Staining intensity was graded as 0 (negative), 1 (weak), 2 (moderate), or 3 (strong), and the positivity percentage was scored as 1 (1%–25%), 2 (26%–50%), 3 (51%–75%), or 4 (76%–100%). For TMA analysis, the final IRS was simplified into four groups: negative (-, score 0–1), weak (+, score 2–4), moderate (++, score 6–8), and strong (+++, score 9–12).

### PPI Network and KEGG pathway analysis

The Search Tool for the Retrieval of Interacting Genes database (STRING) provides information regarding the experimental and predicted protein-protein interactions (PPI). In the present study, we performed PPI analysis to visualize the interactions between proteins. Then KEGG pathway analysis was applied to term the involved processes and pathways of selected genes.

### Cell culture and transfection

U2987 and U2997 glioma cells were of glioblastoma origin and retrieved from a local cell culture bank at the Department of Immunology, Genetics and Pathology, Uppsala University, Sweden. For cell culture, cells were grown in Eagle’s MEM supplemented with 10% fetal bovine serum (FBS) as previously described [32–34]. Lentiviral clones to express either shRNA directed against PTPRZ1 (TRCN0000356375, Target Sequence: CAATCGCATAGGGACGAAATA), OPA1 (CN0000082846, Target Sequence: CCGGACCTTAGTGAATATAAA), or an empty vector control shRNA (SHC001) were obtained from Sigma Aldrich MISSION^®^ (Sigma-Aldrich, USA) and used according to manufacturer’s instructions. DNA plasmids were isolated from the bacterial using plasmid maxiprep kit followed by manufacturer’s instructions (Sigma, USA). Virus production and lentiviral transductions were conducted as previously described [33]. The pCAG-OPA1 plasmid was obtained from Genomeditech (Shanghai, China). GBM cells were transfected with pCAG-OPA1 plasmid using the Lipofectamine 3000 reagent in Opti-MEM for 48 h following the manufacturer’s instructions. Western blotting was used to verify the knockdown effect of PTPRZ1 protein in above cell lines and was conducted as previously described [35].

### Transwell migration assays

Glioma cells (1 × 10^5^) were plated on the top side of Transwell insert (for migration assay) (Corning, USA) in the top chamber of a 12-well cell plate (Corning, USA). Dissociated cells were seeded into chambers, the cells were suspended in medium without serum. Medium supplemented with 10% fetal bovine serum was used as a chemoattractant in the bottom chamber. The cells were incubated at 37℃ for 24 h. The cells that migrated and invaded the lower membrane surface were fixed, stained and counted as previously described [35].

### Zebrafish tumor model

All zebrafish work was performed according to the regulations by the Karolinska Institutet Zebrafish Core Facility. Wild-type AB strains of zebrafish were used for all our experiments. Fertilized (Danio rerio) eggs were incubated at 28 °C in an E3 medium and raised under standard conditions. At 24 h-post-fertilization (hpf), embryos were transferred to an E3 medium containing 0.2 mmol/L 1-phenyl-2-thio-urea (PTU, Sigma) to prevent melanization of embryos. At 48 hpf, embryos were anesthetized with 0.04 mg/ml of tricaine (MS-222, Sigma) prior to further experimentation. For primary cancer models, approximately 500 glioblastoma cells were labeled in vitro with 2 mg/mL of DiI (Sigma) and injected into the perivitelline space of each embryo by an Eppendorf microinjector. After injection, embryos were screened for successful transplantation, and eligible embryos were kept for further experiments. Zebrafish embryos were transferred into 24-well plates (1 embryo/well), and incubated at 33 °C for additional 72 h. Embryos were analysed by fluorescence microscopy (Nikon Eclipse C1), to visualize metastatic foci in the trunk-tail region of each embryo.

### Western blotting

The following primary antibodies were used in Western Blotting experiments: anti-PTPRZ1(1:1000, 610180, BD Biosciences), anti-Tim23 (1:1000; 11802-1-AP, Proteintech), anti-Tom20 (1:1000; 11123-1-AP, Proteintech), anti-OPA1 (1:1000; 612606, BD Biosciences), anti-DRP1 (1:1000, 611113 BD Biosciences), N-Cadherin (1:2000; MCA5698, Bio-Rad), E-Cadherin(1:1000, #3195, CST). After electrophoresis and transfer, PVDF membranes were incubated with primary antibodies on a shaker at 4 °C overnight. Subsequently, the membranes were washed three times with TBST and probed with anti-rabbit or anti-mouse secondary antibodies (1:5000; RD) for 1 h. After three TBST washes, bands were visualized using an enhanced chemiluminescence (ECL) system. Finally, images were captured and grayscale quantification was performed using ImageJ software.

### Reactive oxygen species (ROS) detection and inhibition

Cellular ROS measurements were performed with CM-H2DCFDA (C6827, Invitrogen). Cells were stained with CM-H2DCFDA (1:1000 dilution) for 15 min at 37 °C in the dark, then, Mitotracker red was added for another 15 min. Finally, wash cells with PBS twice and labeled living cells were fixed 4% paraformaldehyde for 10 min examined by confocal microscope or FACS. Mitochondiral ROS was detected by MitoSOX Red Mitochondrial Superoxide Indicators and was performed according to manufacturer’s instructions.

Antioxidant N-Acetyl-L-cysteine (NAC, 50 µM) was added in cell clutures to pre-incubated with GBM cells for 24 h before Transwell migration detections and protein extraction.

### SCENITH assay

To profile the metabolic dependencies and capacities of GBM cells, the SCENITH assay was performed as previously described. In brief, cells were collected for each group and treated with PBS, 2DG (2-Deoxy-D-glucose), Oligomycin A, and double treatment, respectively. Following metabolic inhibition, puromycin was added to each well and incubated for exactly 30 min at 37 °C. After incubation, cells were washed with cold PBS Cells were then fixed and permeabilized using BD Perm/wash buffer (BD Cytofix/Cytoperm™) following manufacturer´s instructions. Intracellular staining of puromycin was done by using Alexa Fluor^®^ 647 anti-Puromycin Antibody (1:500 concentration, Biolegend). Finally, FACS analysis was performed to determine the Puromycin signal for each group. Percentual mitochondrial dependences quantifies how much translation is dependent on oxydative phosphorylation. Data were acquired on a NovoCyte 3000 and analyzed using FlowJo software. The Metabolic Dependency and Capacity were calculated based on the Geometric Mean Fluorescence Intensity (gMFI) of the puromycin signal using the following formulas: Glucose dependence (%): 100 x ((MFI PBS – MFI 2-DG)/(MFI PBS – MFI BOTH)); Fatty acid oxidation and amino acid oxidation capacity (%): 100% - Glucose dependence; Mitochondrial dependence (%): 100 x ((MFI PBS – MFI OLIGO)/(MFI PBS – MFI BOTH))Glycolytic capacity (%): 100% - Mitochondrial dependence.

### Immunofluorescent staining

Tyramide Signal Amplification (TSA) method was used for multiplex immunofluorescence (mIF) staining of paraded GBM tissues with antibody against PTPRZ1 (1:100, 55125-1-AP, Proteintech), OPA1 (1:100, 27733-1-AP, Proteintech) and N-Cadherin (1:200, ab18203) was performed according to manufacturer´s instructions. Cy5 Goat Anti-Rabbit IgG H&L secondary antibody (1:200, abcam), Cy3 Goat Anti-Rabbit IgG H&L secondary antibody (1:200, abcam) and Alexa-Fluor 488 Goat Anti-Rabbit IgG H&L secondary antibody (1:500, abcam, USA), Goat Anti-Rabbit IgG H&L (HRP) (1:2000, abcam) were used for fluorescent multiplex-staining. ImageJ was used for the quantification and visualization of colocalized signals.

### Statistical analysis

Statistical analyses were performed using GraphPad Prism 11. Kaplan–Meier survival analysis was used to estimate the survival distributions, and the log-rank test was used to assess the statistical significance between stratified survival groups. Normality of data distribution was assessed using the Shapiro–Wilk test. When data followed a normal distribution, Student’s t-test was used to determine differences between each two groups. One-way analysis of variance (ANOVA) was performed, followed by Tukey’s post hoc test for multiple comparisons. All data are presented as the mean ± standard error. P-values < 0.05 were considered statistically significant.

## Result

### PTPRZ1 is highly expressed in glioma tissues and correlated with worse OS

**Fig. 1:**
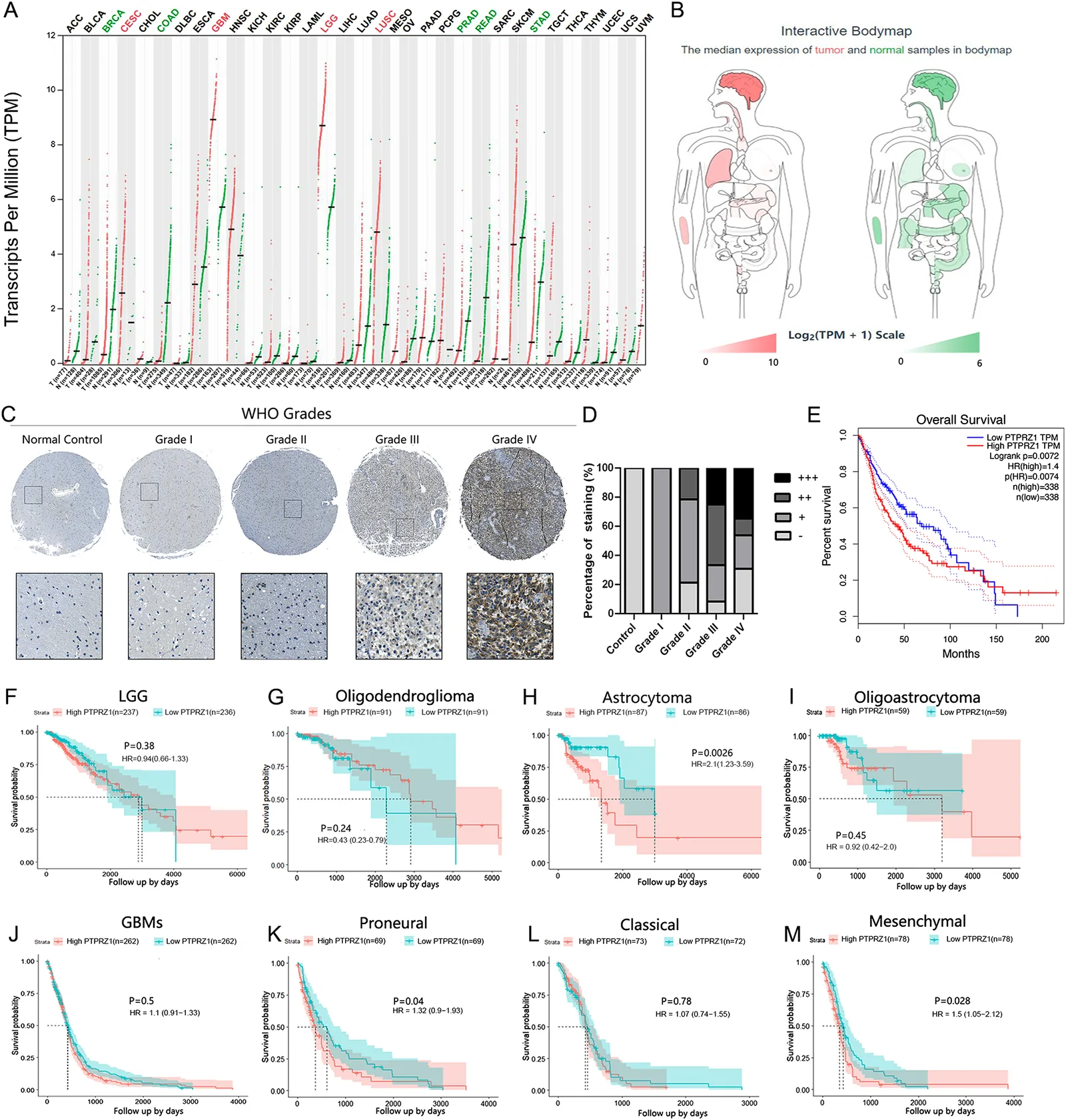
The expression of PTPRZ1 in gliomas.** (A-B)** GEPIA analysis reveals the mRNA expression of PTPRZ1 in pan cancers and its paired control tissues. **(C-D)** The immunohistochemistry and quantitative analysis of glioma tissue microarray reveal the protein level of PTPRZ1 in different grades of glioma and control tissue. **(E)** KM survival plot of PTPRZ1 in glioma patients from GEPIA TCGA dataset. **(F-M)** The survival plot of PTPRZ1 in LGG and GBM. **(F)** LGG **(G)** Oligodendroglioma **(H)** Astrocytoma **(I)** Oligoastrocytoma **(J)** GBMs **(K)** Proneural **(L)** Classical **(M)** Mesenchymal GBM subtypes. Median value was used to divide patients into Low and High PTPRZ1 expression group

**Fig. 2:**
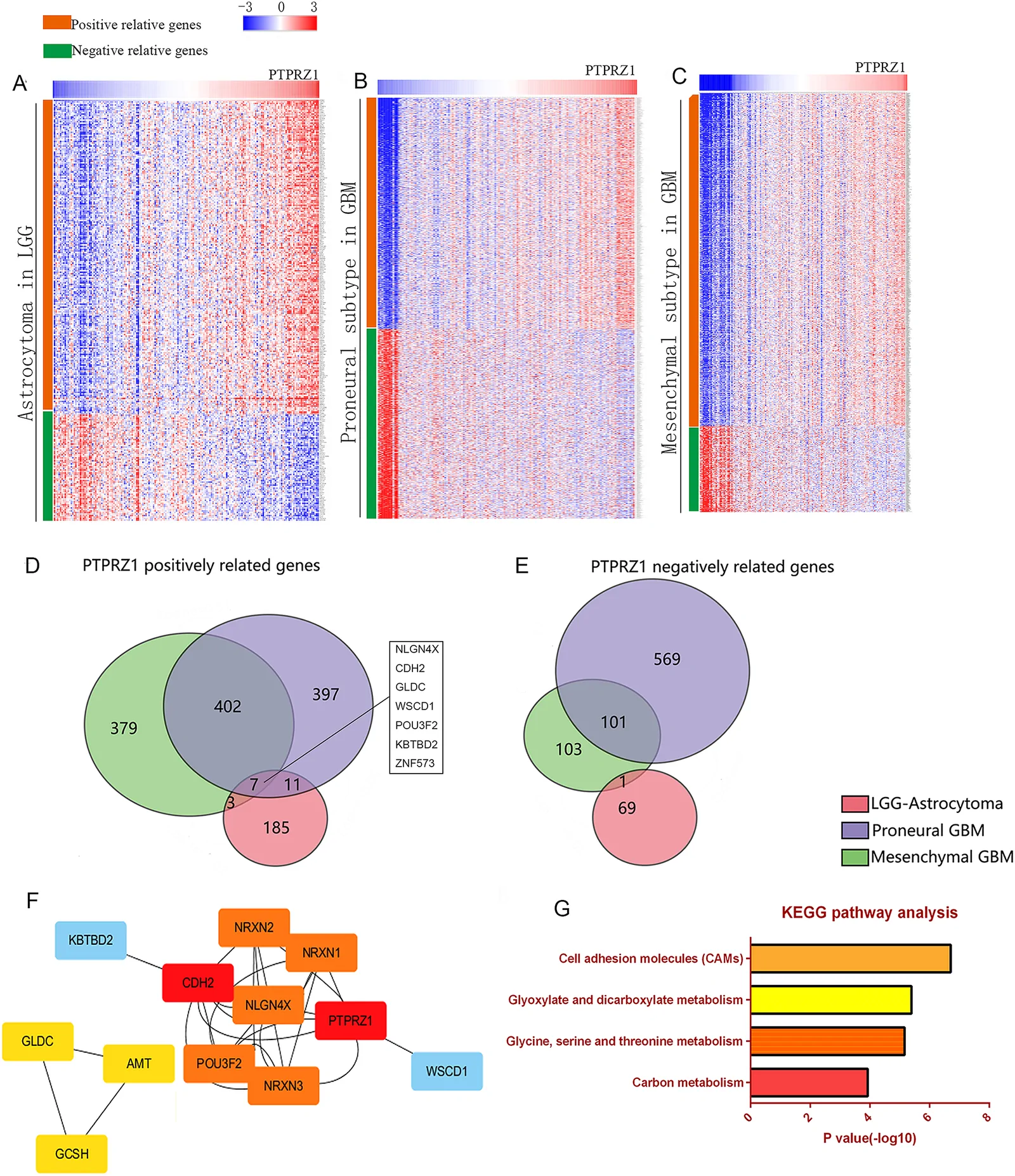
Pearson analysis and Venn diagram compared correlated genes in LGG-astrocytoma, Proneural and Mesenchymal GBMs. Pearson assay displayed the PTPRZ1 most closely related genes (|R|≥0.5) in (**A**) LGG-astrocytoma, (**B**) Proneural and (**C**) Mesenchymal GBM subgroups and visualized through heatmap visualization. (**D**)Venn diagram comparing the PTPRZ1 positively associated genes between LGG-astrocytoma, Proneural and Mesenchymal GBMs. (**E**) Venn diagram comparing the PTPRZ1 positively associated genes between LGG-astrocytoma, Proneural and Mesenchymal GBMs. (**F**) PPI analysis of the PTPRZ1 common associated genes (NLGN4, POU3F2, WSCD1, CDH2, GLDC, ZNF573, KBTBD2) and their interactors in gliomas. (**G**) KEGG analysis of the PPI result and ranked by p value

To evaluate the expression of PTPRZ1 among tumors, we downloaded the mRNA expression profile of PTPRZ1 in pan cancers from GEPIA (http://gepia.cancer-pku.cn/) (Fig. 1A-B). Notably, we found GBM and LGG (Lower grade glioma, Grade 2 and Grade 3 gliomas) own the highest PTPRZ1 expression compared to all the other tumors. Then, we applied immunohistochemistry to the tissue microarray (Supplementary Fig. 1) (different grades of glioma tissues from 55 patients) to examine the protein level of PTPRZ1 in glioma. The results also demonstrated that PTPRZ1 is commonly expressed in glioma tissues, and increased immunostaining of PTPRZ1 was found in high grade glioma patients (Grade 3 glioma and GBM) (Fig. 1C-D). To explore the influence of PTPRZ1 expression on patient survival, we retrieved the clinical data of lower grade glioma and GBM from TCGA dataset. We analyzed the prognosis of glioma patients by Kaplan–Meier method and using the median value of PTPRZ1 expression as the threshold. When all glioma patients were analyzed together, high PTPRZ1 expression showed association with poor OS time (Fig. 1E). When cohort was subdivided into specific pathological and molecular subgroups (Fig. 2F-M), significant difference between PTPRZ1-low group and PTPRZ1-high group in OS time can be observed in astrocytoma of LGG, proneural GBM and mesenchymal GBM (Fig. 2H, K, M). Findings suggest that PTPRZ1 is highly expressed in glioma and may functions as a key driver of malignant transition.

### PTPRZ1 correlates with cell adhesion molecules in gliomas

To delineate the regulatory pattern of PTPRZ1 across different glioma grades and subtypes, including LGG-astrocytoma as well as proneural and mesenchymal GBMs, we performed a genome-wide Pearson correlation analysis. Genes significantly correlated with PTPRZ1 (Pearson |R| > 0.5) in each cohort were identified and visualized using heatmaps (Fig. 2A-C). A Venn diagram analysis was subsequently employed to identify overlapping genes across the three cohorts (Fig. 2D-E). Intriguingly, only seven genes (NLGN4X, CDH2, GLDC, WSCD1, POU3F2, KBTBD2, ZNF573) positively correlated with PTPRZ1 were found to overlap across all subtypes, while no common negatively correlated genes were identified. To further elucidate the biological processes associated with PTPRZ1, we performed Protein-Protein Interaction (PPI) and KEGG pathway enrichment analyses on these seven coregulated genes. The results demonstrated a robust association between PTPRZ1 and cell adhesion molecules (CAMs), which are well-documented mediators of tumor migration and invasion (Fig. 2F-G).

### PTPRZ1 promotes mesenchymal transition and glioma cell mobility

**Fig. 3:**
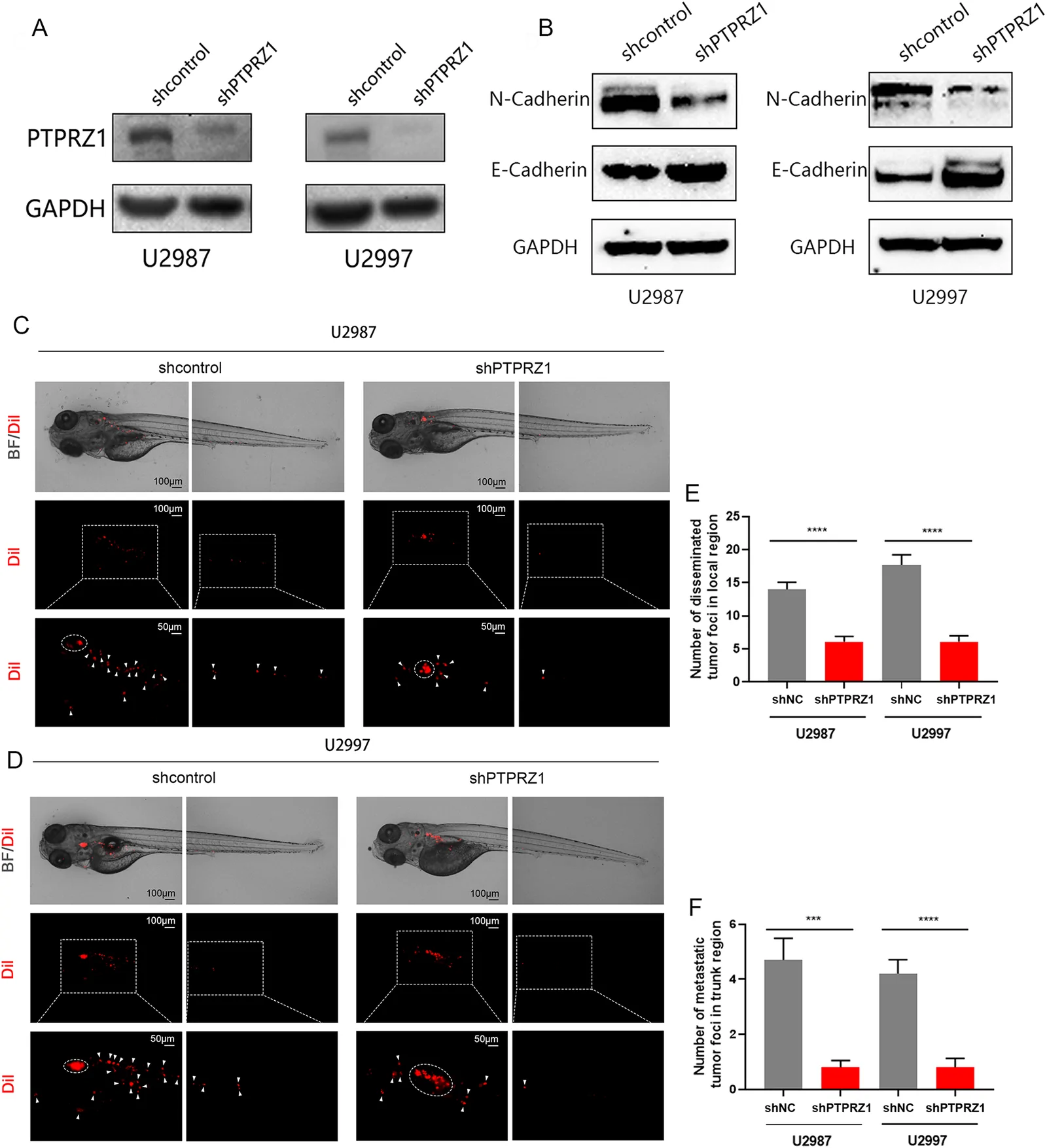
PTPRZ1 is essential for glioma migration.** (A-B)** Western blotting analysis of PTPRZ1, N-cadherin and E-cadherin expression in shcontrol and shPTPRZ1 groups from U2987 and U2997 glioma cells. **(C-D)** The zebrafish tumor migration model was applied to explore the migration ability of U2987 and U2997 cells in shcontrol and shPTPRZ1 groups. **(E-F)** Quantification of the number of disseminated foci in the local region and metastatic foci in the trunk region

To experimentally validate the role of PTPRZ1 in glioma motility, we established stable PTPRZ1-knockdown glioma cell lines (U2987 and U2997) via lentiviral transfection, with knockdown efficiency confirmed by western blotting (Fig. 3A). Phenotypically, PTPRZ1 depletion was associated with a clear shift in EMT markers, characterized by decreased N-cadherin expression and increased E-cadherin expression (Fig. 3B).

To assess the impact of PTPRZ1 on tumor migration in vivo, we employed a zebrafish xenograft model. Quantification of tumor dissemination revealed that the shPTPRZ1 group had significantly fewer disseminated tumor foci in the local cranial region and fewer metastatic foci in the trunk compared to the control group (Fig. 3C-F). Collectively, these multi-level evidence confirm that PTPRZ1 is a master regulator of the invasive phenotype in both LGG and GBM, in a manner involving modulation of the cell adhesion and EMT programs.

### PTPRZ1 is particularly expressed in tumor infiltrating area

**Fig. 4:**
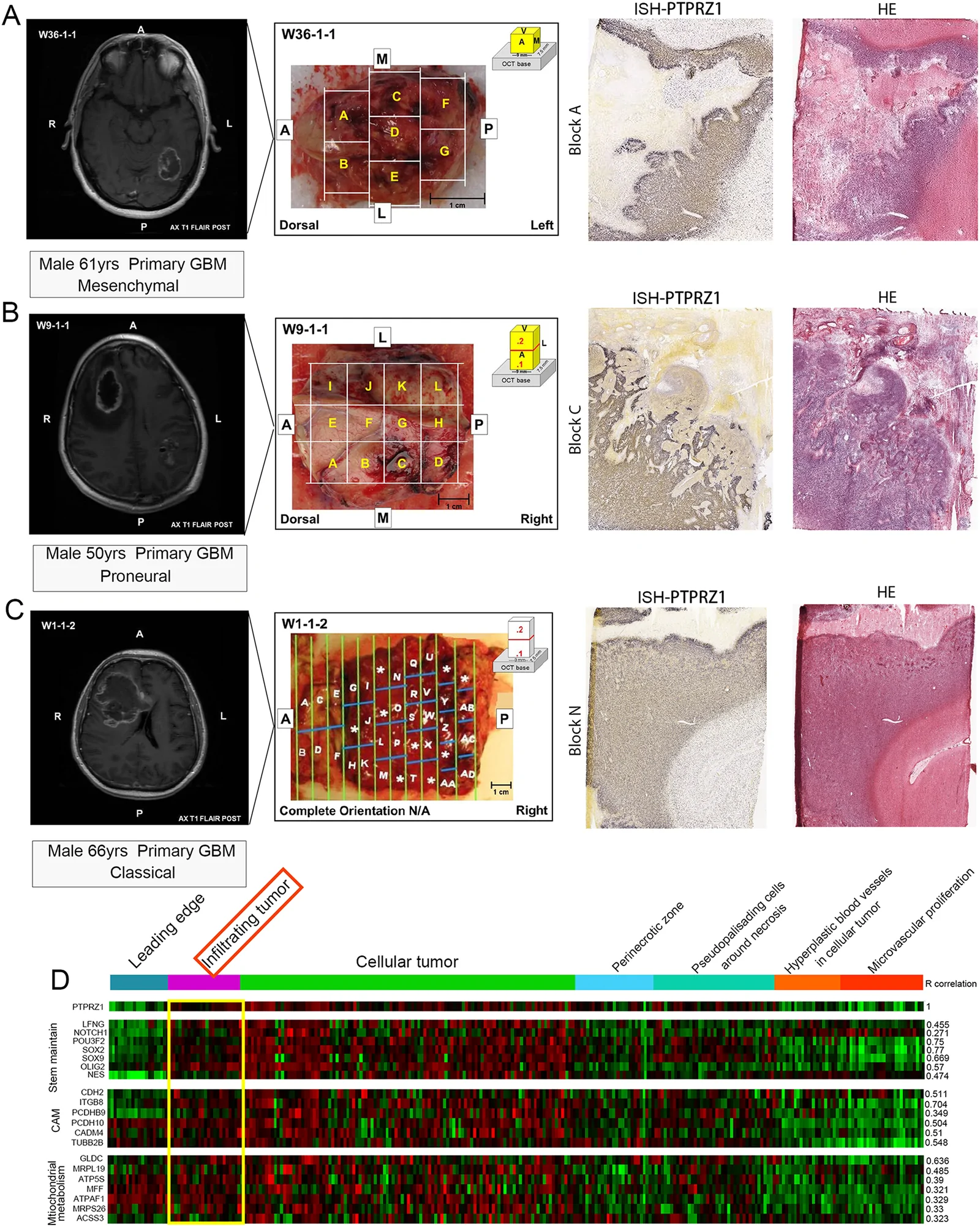
Spatial mapping and molecular characterization of PTPRZ1 in GBM.** (A-C)** Representative multi-modal imaging and histopathological analysis of three primary GBM cases across molecular subtypes: **(A)** Mesenchymal (Male, 61 yrs), **(B)** Proneural (Male, 50 yrs), and **(C)** Classical (Male, 66 yrs) from Ivy GBM dataset. **(D)**The expression of PTPRZ1 and other molecular markers in RNA sequencing-based regional subtypes

To better understand the role of PTPRZ1 in the rapid progression and invasion of glioblastoma, we first analyzed its spatial expression patterns using the Ivy Glioblastoma Atlas Project (Ivy GAP) dataset. By integrating navigated surgical MRI imaging with in situ hybridization (ISH) staining, we observed that PTPRZ1 is predominantly expressed in the infiltrating zones situated between the tumor core and normal brain tissue(Fig. 4A-C). Spatial RNA-sequencing data further suggested that PTPRZ1 expression strongly correlates with stemness markers, cell adhesion molecules (CAMs), and mitochondrial metabolism within both the infiltrating and cellular tumor regions (Fig. 4D).

**Fig. 5:**
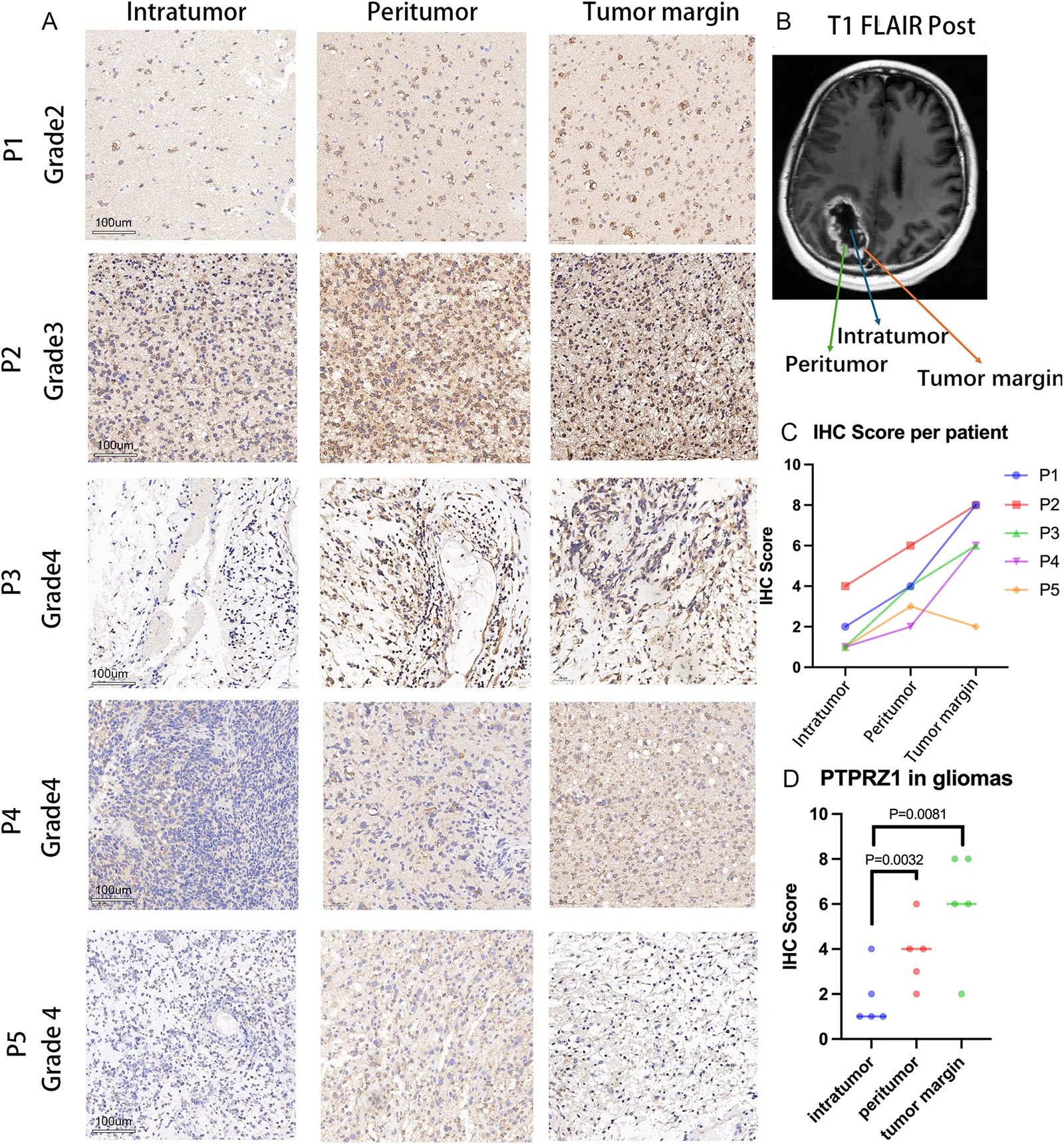
Elevated expression of PTPRZ1 at the invasive tumor margin across human glioma grades.** (A)** Representative images of immunohistochemical (IHC) staining for PTPRZ1 within matched spatially distinct regions from five glioma patients. **(B)** T1-weighted MRI approximate surgical sampling locations corresponding to the spatial regions in (**A**): intratumoral core (blue), peritumoral region (green), and invasive tumor margin (orange). **(C)** PTPRZ1 expression within individual patients. Line graph showing the PTPRZ1 IHC Score for each tissue section across the Intratumor, Peritumor, and Tumor margin regions for all five patients (P1–P5). (**D**) Collective quantitative analysis of PTPRZ1 expression in gliomas. Dot plot summarizing the IHC Scores for the three spatial regions across all analyzed tumor tissues (RM-ANOVA test, * *P* < 0.05, **P < 0.01)

To validate these bioinformatic predictions, we analyzed an internal clinical cohort of glioma samples, which were spatially categorized into intratumoral, peritumoral, and tumor margin regions for each patient (Fig. 5A-B). Immunohistochemical (IHC) staining confirmed that PTPRZ1 expression is significantly elevated in the peritumor and tumor margin areas compared to the tumor core (Fig. 5C-D). These findings reinforce the potential involvement of PTPRZ1 in tumor migration and its regulatory role in cell adhesion and the epithelial-to-mesenchymal transition (EMT) process during GBM infiltration.

### PTPRZ1 promotes glioma migration by suppressing ROS production

**Fig. 6:**
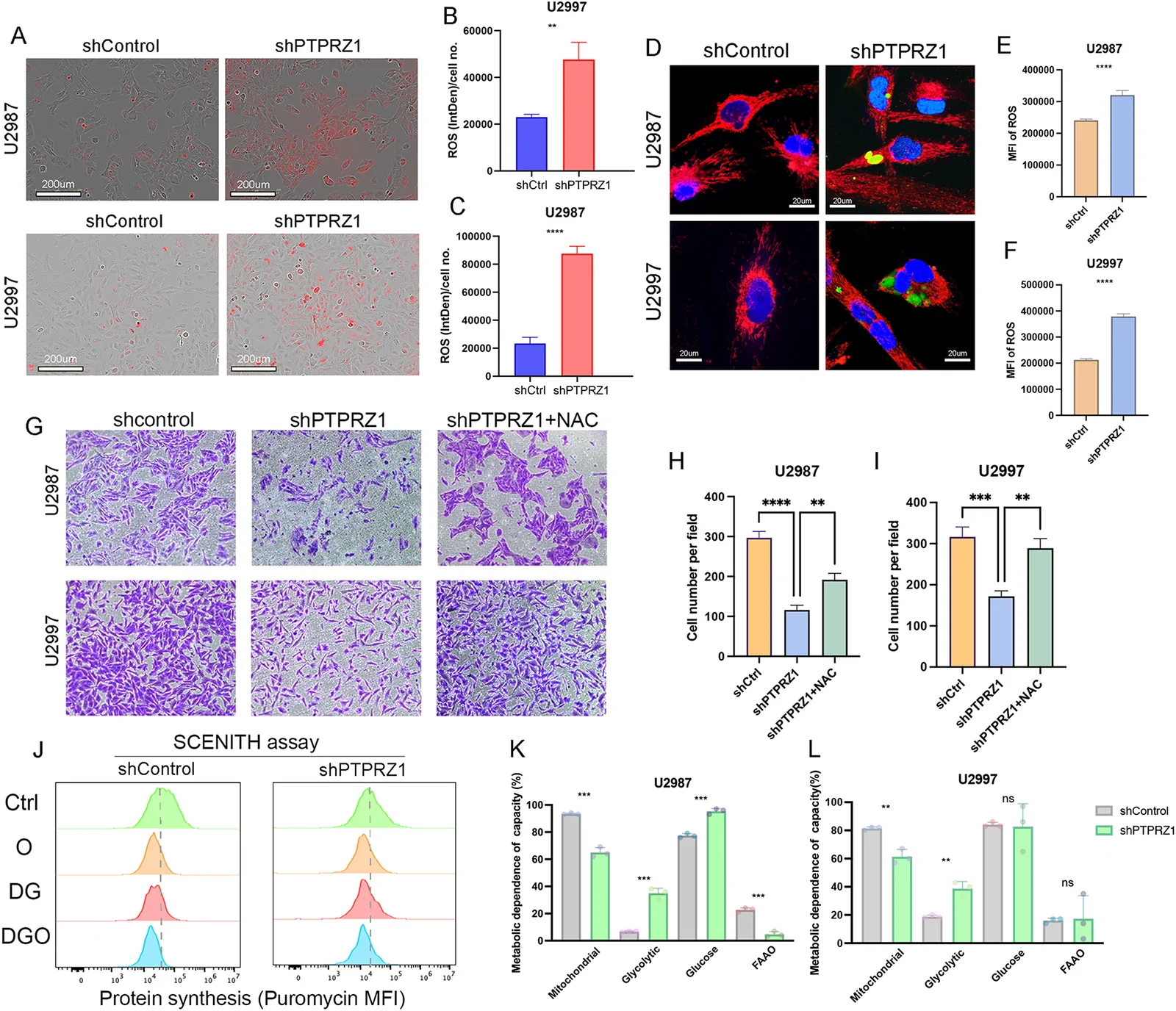
Loss of PTPRZ1 induces mitochondrial ROS accumulation and dysfunction.** (A)** Representative Incucyte confocal images showing mitochondrial superoxide levels (detected by MitoSOX) in control and PTPRZ1-deficient U2987 and U2997 cells. **(B-C)** Quantification of mitochondrial ROS in U2987 and U2997 cells with or without PTPRZ1 knockdown. (**D**) Representative confocal images showing mitochondrial superoxide levels (CM-H2DCFDA, Green) in control and PTPRZ1-deficient U2987 and U2997 cells. **(E-F)** Quantification of cellular ROS (CM-H2DCFDA, Green)in U2987 and U2997 cells by FACS. **(G)** Transwell assay showing migrated U2987 and U2997 cells in shcontrol, shPTPRZ1 and shPTPRZ1 + NAC groups. **(H-I)** Quantitative analysis of transwell migration assay in U2987 and U2997 glioma cells respectively. **(J)** Representative histograms of puromycin incorporation (MFI), as measured by the SCENITH assay, under PBS (Control), Oligmycin, 2DG, 2DG+Oligmycin in vitro culture. **(K-L) **Bar plot (*n* = 3) showing the metabolic profile of U2987 and U2997 cells with Control or PTPRZ1KD obtained by SCENITH

Based on our previous findings, we hypothesized that PTPRZ1 might be involved in maintaining mitochondrial metabolism. To test this, we measured both mitochondrial and cellular ROS levels using fluorescence probes. Confocal imaging analysis of MitoSOX red fluorescent mitochondrial superoxide indicator suggested that markedly enhanced ROS production upon knockdown of PTPRZ1 in GBM cells (Fig. 6A-C). Meanwhile, confocal imaging of CM-H2DCFDA (green fluorescence) also showed a significantly increased the accumulation of general ROS in PTPRZ1 knockdown U2987 and U2997 cells (Fig. 6D), this observation was quantified by flow cytometry (MFI) (Fig. 6E-F). Furthermore, SCENITH metabolism assays indicated that PTPRZ1 deficiency triggered a metabolic shift from mitochondrial oxidative respiration to a glycolytic-dependent state in U2987 and U2997 cells (Fig. 6G-I). Importantly, Transwell assays demonstrated that the suppression of glioma cell migration induced by PTPRZ1 knockdown could be rescued by pre-treatment with 3mM antioxidant NAC (a ROS inhibitor) (Fig. 6J-L). Collectively, these results suggest that PTPRZ1 is essential for maintaining mitochondrial function and limiting ROS accumulation, which ultimately sustains glioma cell mobility.

### PTPRZ1 suppressed ROS production by maintaining mitochondrial fusion

**Fig. 7:**
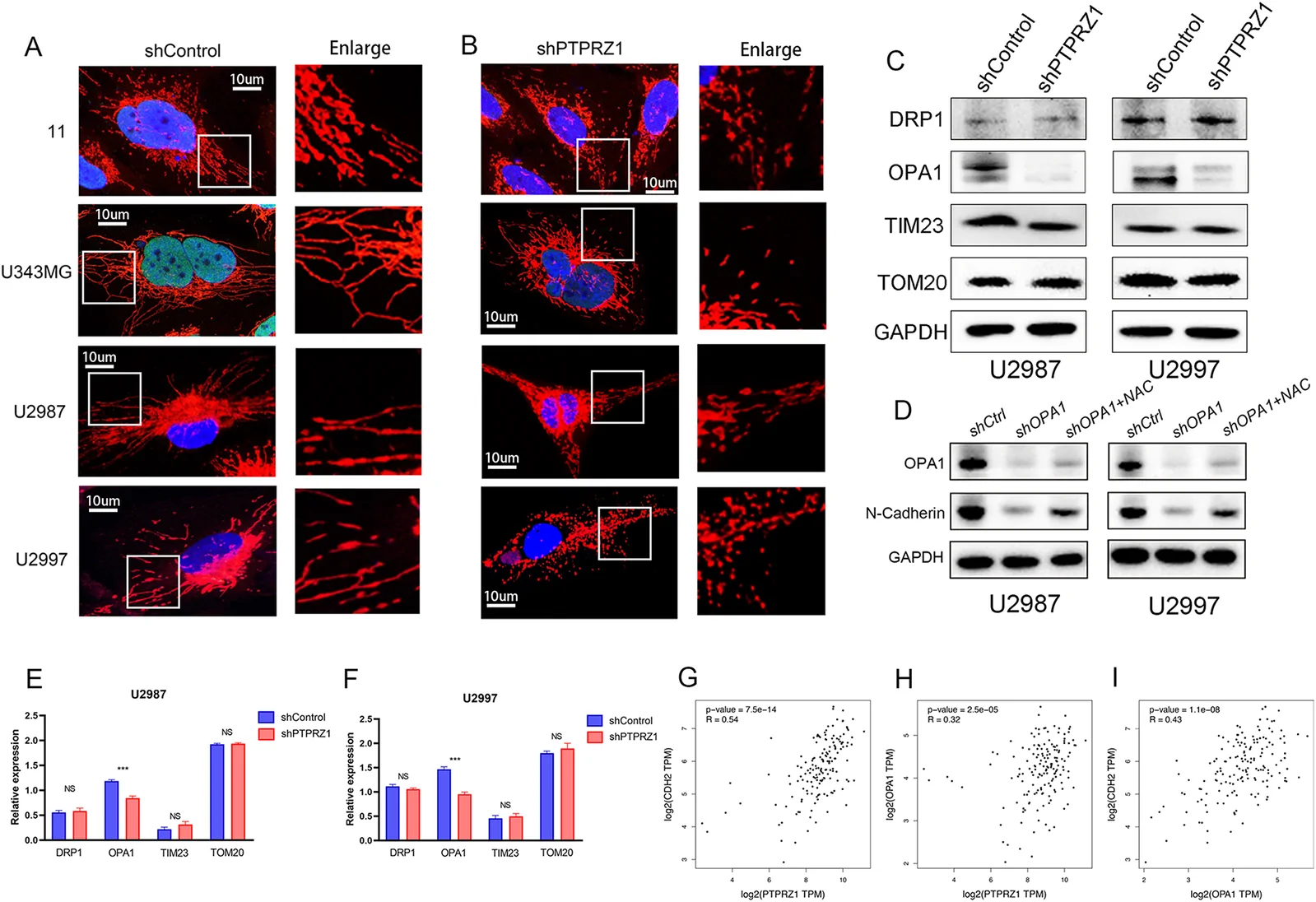
PTPRZ1 maintains N-cadherin expression by regulating OPA1-mediated mitochondrial dynamics.** (A-B)** Confocal imaging of mito-tracker red stained GBM cells (#11, U343MG, U2997, U2987) in control and shPTPRZ1 groups. **(C)** Western blots showing protein expression of DRP1 OPA1 TIM23 and TOM20 after PTPRZ1 knockdown in U2987 and U2997 cells. **(D)** Western blots showing expression of OPA1 and N-Cadherin with shCtrl shOPA1 and shOPA1 + NAC treatment in U2987 and U2997 cells. **(E-F)** Quantification of protein expression **(C)** was normalized to GAPDH as a loading control. **(G-I)** Correlation analysis of PTPRZ1, OPA1 and N-Cadherin mRNA expression in TCGA GBM samples

**Fig. 8:**
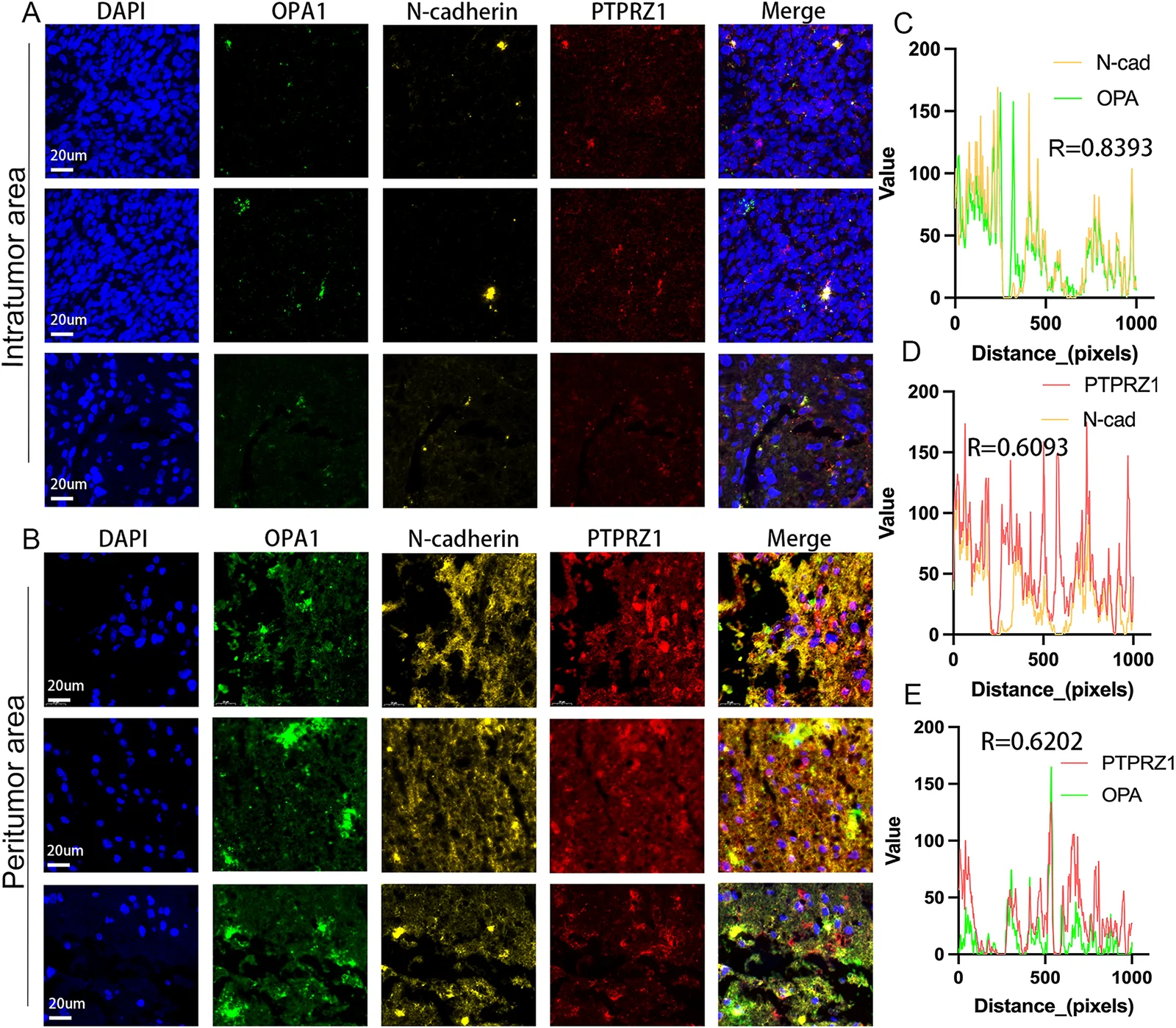
PTPRZ1, OPA1 and N-Cadherin are enriched in peritumoral area. (**A**-**B**) Representative immunofluorescence (IF) images of OPA1 (green), N-cadherin (yellow), and PTPRZ1 (red) in the (**A**) intratumor area and (**B**) peritumor area of GBM clinical specimens. Cell nuclei were counterstained with DAPI (blue). Scale bars = 20 μm. (**C**-**E**) Co-localization Pearson analysis of the expression of indicated protein pairs across the peritumoral tissue sections. (OPA1 and N-cad, R = 0.8393; PTPRZ1 and N-cad, R = 0.6093; PTPRZ1 and OPA1, R = 0.6202)

To further elucidate the mechanism by which PTPRZ1 regulates mitochondrial activity and its association with cancer cell migration, we examined mitochondrial morphology using MitoTracker Red staining across multiple primary glioma cell cultures (#11, U343MG, U2987, U2997). Strikingly, knockdown of PTPRZ1 induced a phenotypic transition from elongated to highly fragmented and fissionary mitochondria (Fig. 7A-B). Western blotting revealed that PTPRZ1 knockdown led to a significant decrease in OPA1 expression, while mitochondrial structural proteins and the fission-related protein DRP1 remained unaffected (Fig. 7C, E-F). Furthermore, OPA1 silencing resulted in a significant reduction in N-cadherin (CDH2) expression, and this downregulation could be partially rescued by pre-treatment with the antioxidant NAC. (Fig. 7D). Collectively, these findings suggest that PTPRZ1 maintains CDH2 expression by upregulating OPA1, which preserves mitochondrial function and reduces ROS accumulation. To further validate the regulatory role of the PTPRZ1-OPA1-N-cadherin axis in glioma migration, we performed Pearson correlation analysis on the TCGA-GBM dataset, which revealed a strong positive correlation among PTPRZ1, OPA1, and CDH2 (N-cadherin) mRNA expression (Fig. 7G–I). This association was further confirmed by multi-fluorescence staining of GBM intratumoral and peritumoral tissues (Fig. 8A–B), which demonstrated the consistent co-enrichment and spatial overlap of these markers specifically within the peritumoral regions (Fig. 8C–E).

### PTPRZ1 promotes GBM migration via OPA1/ROS/N-Cadherin axis

**Fig. 9:**
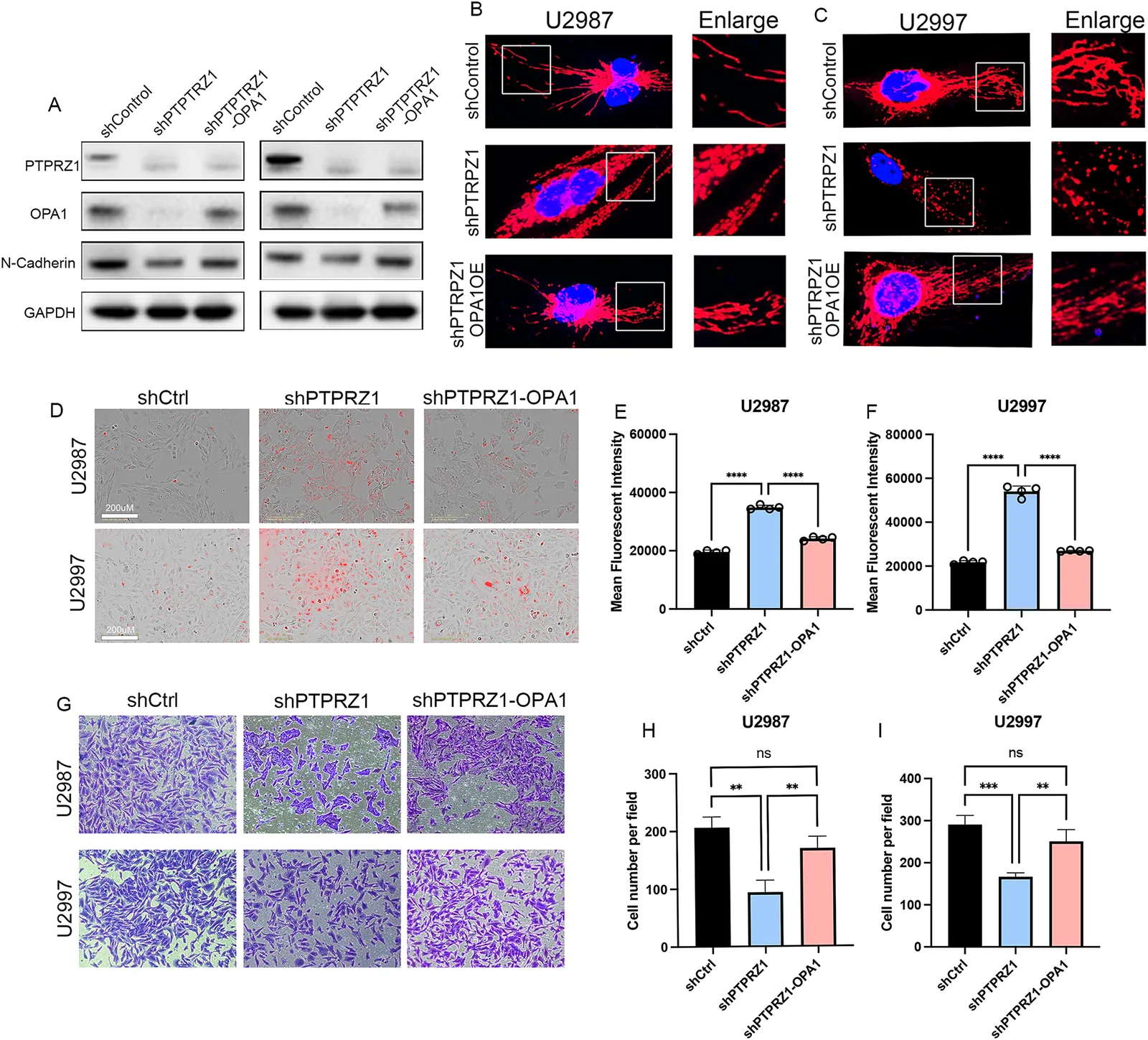
OPA1 re-expression restores mitochondrial homeostasis and migratory potential in PTPRZ1-deficient GBM cells. (**A**) Western blotting analysis of PTPRZ1, OPA1, N-Cadherin expression in shcontrl, shPTPRZ1 and shPTPRZ1-OPA1 overexpressed groups from U2987 and U2997 GBM cells. (**B**–**C**) Representative images of mitochondrial morphology in U2987 (B) and U2997 (**C**) cells. Mitochondria were stained with a red fluorescent dye. Scale bars: 10 μm (enlarged insets). (**D**–**F**) Evaluation of mitochondrial reactive oxygen species (ROS) levels in indicated GBM cells. **(D) **Representative images of ROS staining. (**E**–**F**) Quantification of mean fluorescent intensity in U2987 and U2997 cells measured by flow cytometry. (One Way ANOVA test, Data are presented as mean ± SD, ****p < 0.0001) (**G**–**I**) Transwell migration assay of indicated GBM cells. (**G**) Representative images of migrated cells stained with crystal violet. (**H**–**I**) Quantification of migrated cell numbers per field in U2987 and U2997 cells. (One-Way ANOVA test, Data are presented as mean ± SD, p < 0.01, ***p < 0.001, ns = not significant)

To explore whether OPA1 acts as a downstream effector of PTPRZ1 in regulating GBM cell motility, we performed rescue experiments by re-expressing OPA1 in PTPRZ1-knockdown (KD) cells. Western blot analysis revealed that OPA1 re-expression successfully restored N-cadherin (CDH2) protein levels that had been suppressed by PTPRZ1-KD in both U2987 and U2997 cell lines (Fig. 9A). Fluorescence imaging of mitochondrial morphology showed that PTPRZ1 depletion led to severe mitochondrial fragmentation, which was rescued by OPA1 re-expression, restoring the elongated, tubular mitochondrial dynamics (Fig. 9B-C). Furthermore, this morphological restoration was accompanied by a significant mitigation of mitochondrial oxidative stress, as evidenced by the decreased mitochondrial ROS levels in OPA1-overexpressed cells compared to PTPRZ1-KD group (Fig. 9D–F). Functionally, transwell migration assays demonstrated that the re-expression of OPA1 significantly rescued the migration defects caused by PTPRZ1 depletion (Fig. 9G–I). Collectively, these findings combined with our previous data support that PTPRZ1 promotes GBM cell motility through the PTPRZ1/OPA1/ROS/N-cadherin axis.

## Discussion

The rapid progression and diffuse infiltration of glioblastoma (GBM) remain a formidable therapeutic challenge [36]. Recent studies have highlighted PTPRZ1 as a master regulator of the invasive phenotype during the development of glioblastoma [26, 37], the precise molecular mechanisms governing its sustained activity within these invasive niches remain largely elusive.

In the present study, we identified PTPRZ1 as a driver of poor prognosis in gliomas, particularly in grade 2/3 astrocytomas as well as proneural and mesenchymal GBMs. Given the ‘secondary glioblastoma’ hypothesis—which posits that GBMs evolve from low-grade astrocytomas—and the well-documented shift from a proneural to a mesenchymal molecular pattern upon recurrence, indicating that PTPRZ1 may serve as a crucial mediator of this malignant evolution [38, 39]. By integrating TCGA and Ivy GAP datasets with our internal clinical cohort, we demonstrated that PTPRZ1 is significantly enriched in the peritumoral and margin areas, where it correlates with markers of cell adhesion. This molecular mechanism was further validated by the shift from N-cadherin to E-cadherin in PTPRZ1-deficient cells. Furthermore, in a zebrafish xenograft model, PTPRZ1 depletion significantly impaired local invasion and distant dissemination.

The dynamic balance of mitochondrial metabolism and oxidative stresses is essential for cancer cell fate determination and cell migration [40, 41]. Our data reveal that PTPRZ1 sustains an elongated mitochondrial network by maintaining the expression of mitochondrial fusion protein OPA1. The transition from elongated to fragmented mitochondria upon PTPRZ1 knockdown highlights a critical regulatory axis governing mitochondrial dynamics. This morphological shift is accompanied by metabolic reprogramming from oxidative phosphorylation toward glycolytic dependence and elevated ROS accumulation. While moderate levels of ROS act as critical signaling mediators for tumorigenesis [42], excessive accumulation triggers irreversible oxidative damage and cellular dysfunction [43]. These results indicating that PTPRZ1-maintained OPA1 expression preserve mitochondrial function under the metabolic stress associated with tumor infiltration. The functional impact of this PTPRZ1/OPA1/ROS axis is further confirmed by its regulation of the mesenchymal transition. We observed that the suppression of N-cadherin (CDH2) following OPA1 knockdown could be effectively rescued by the antioxidant NAC, identifying ROS as the key intermediate that destabilizes the PTPRZ1 mediated migratory machinery. Moreover, re-expressing OPA1 in PTPRZ1-KD cells effectively restores mitochondrial elongation, attenuates mitochondrial ROS production, and consequently rescues migratory capacity. These findings suggest that PTPRZ1 maintains the GBM cell mobility by preserving OPA1-mediated mitochondrial dynamics and restricting excessive ROS accumulation.

In conclusion, our study demonstrates a regulatory network that PTPRZ1 promotes glioma migration by sustaining an OPA1-dependent mitochondrial fusion program. This program mitigates ROS-induced oxidative stress, thereby maintaining N-cadherin expression and driving the invasive phenotype. Given its enrichment in the peritumoral invasive niche, targeting the PTPRZ1/OPA1 axis may serve a promising therapeutic strategy to curb the relentless spread of glioblastoma.

## Supplementary Information

Below is the link to the electronic supplementary material.


Supplementary Material 1


## Data Availability

Publicly available datasets analyzed in this study can be found in The Cancer Genome Atlas (TCGA) repository ((https://xena.ucsc.edu/)) and the Ivy Glioblastoma Atlas Project (http://glioblastoma.alleninstitute.org/). Additional data and materials related to this study are available from the corresponding author upon reasonable request.

## Abbreviations

GBM: Glioblastoma
TCGA: The Cancer Genome Atlas
PTPRZ1: Protein Tyrosine Phosphatase, Receptor Type Z1
IDH1/2: Isocitrate dehydrogenase1/2
OPCs: Oligodendrocyte precursor cells
RTKs: Receptor tyrosine kinases
PTN: Pleiotrophin
LGG: Lower grade glioma
GSEA: Gene Set Enrichment Analysis
CNS: Central nervous system
RTP: Receptor tyrosine phosphatase
CAMs: Cell adhesion molecules
CORUM: The comprehensive resource of mammalian protein complexes
