# Supplementary material for: Spatial enrichment of PTPRZ1 in the peritumoral niche drives glioblastoma migration via the OPA1/ROS/CDH2 axis
Source: J Transl Med. 2026 Aug 26;24:1103. doi: 10.1186/s12967-026-08884-7 (PMC13523290; doi:10.1186/s12967-026-08884-7)
Supplement: Supplementary file 1 [file 12967_2026_8884_MOESM1_ESM.docx]

**Supplementary Fig1**

**SFig1.Tissue microarray (TMA) analysis of PTPRZ1 expression in glioma.**

**(A)** Representative image of the TMA slide used for immunohistochemical staining of PTPRZ1. **(B)** Detailed clinicopathological characteristics of the patient cohort included in the TMA, including case ID, tumor grade, pathological diagnosis, age, gender, and tumor location.

**SFig2.** **PTPRZ1 knockdown does not alter mitochondrial mass, cell cycle progression, or proliferation in glioma cells.**

**(A)** Quantification of mitochondrial mass in U2987 and U2997 cells expressing shCtrl or shPTPRZ1, measured by mean fluorescence intensity. Data are presented as mean ± SEM (ns, not significant). (B) Cell cycle distribution analysis of U2987 and U2997 cells transduced with shCtrl or shPTPRZ1. The percentage of cells in G1, S, and G2 phases is shown. (C) Proliferation assays for U2987 (left) and U2997 (right) cells, comparing control and shPTPRZ1 groups across four time points (D1–D4), as measured by fluorescence (560/590 nm).

**Supplementary Fig2**
